# Stomatin Inhibits Pannexin-1-Mediated Whole-Cell Currents by Interacting with Its Carboxyl Terminal

**DOI:** 10.1371/journal.pone.0039489

**Published:** 2012-06-29

**Authors:** Haiying Zhan, Craig S. Moore, Bojun Chen, Xin Zhou, Xin-Ming Ma, Kumiko Ijichi, Michael V. L. Bennett, Xue-Jun Li, Stephen J. Crocker, Zhao-Wen Wang

**Affiliations:** 1 Department of Neuroscience, University of Connecticut Health Center, Farmington, Connecticut, United States of America; 2 Center on Aging, University of Connecticut Health Center, Farmington, Connecticut, United States of America; 3 Department of Neuroscience, Albert Einstein College of Medicine, Bronx, New York, United States of America; Dalhousie University, Canada

## Abstract

The pannexin-1 (Panx1) channel (often referred to as the Panx1 hemichannel) is a large-conductance channel in the plasma membrane of many mammalian cells. While opening of the channel is potentially detrimental to the cell, little is known about how it is regulated under physiological conditions. Here we show that stomatin inhibited Panx1 channel activity. In transfected HEK-293 cells, stomatin reduced Panx1-mediated whole-cell currents without altering either the total or membrane surface Panx1 protein expression. Stomatin coimmunoprecipitated with full-length Panx1 as well as a Panx1 fragment containing the fourth membrane-spanning domain and the cytosolic carboxyl terminal. The inhibitory effect of stomatin on Panx1-mediated whole-cell currents was abolished by truncating Panx1 at a site in the cytosolic carboxyl terminal. In primary culture of mouse astrocytes, inhibition of endogenous stomatin expression by small interfering RNA enhanced Panx1-mediated outward whole-cell currents. These observations suggest that stomatin may play important roles in astrocytes and other cells by interacting with Panx1 carboxyl terminal to limit channel opening.

## Introduction

Pannexin-1 (Panx1) is a mammalian homologue of the invertebrate gap junction proteins, innexins [1], [2]. It is almost ubiquitously expressed in mammalian tissues [1], [3] and forms membrane channels implicated in a variety of physiological or pathological functions, including ATP release [4]–[7], propagation of Ca^2+^ waves between cells [8], epileptiform seizure activity [9], [10], activation of the inflammasome [11], and recruitment of macrophages to apoptotic cells by releasing “find-me” signals [12]. The Panx1 channel has a large single-channel conductance (∼550 pS) [13], [14] and allows the passage of relatively large molecules such as ATP, arachidonic acid derivatives, and fluorescent dyes [15]. While opening of the channel is necessary for its physiological functions, uncontrolled opening may lead to a rapid depletion of ionic gradients and cell death [13]. Thus, the Panx1 channel likely exists mainly in the closed state under physiological conditions. A variety of factors have been shown to cause the opening of Panx1 channels, including membrane depolarization [1], [16], elevation of intracellular [Ca^2+^] [8], mechanical stress [4], [14], activation of P2Y purinergic receptors by extracellular ATP [8], apoptosis [6], [12], NMDA receptor activation [9], and ischemic or hypoxic conditions [13], [17]. However, relatively little is known about the mechanisms that close the channel. One study shows that ATP released into the extracellular space through the Panx1 channel may inhibit the channel activity and thus serve as a brake to prevent further release [18], [19]. Another study shows that the Panx1 channel is inhibited by the reducing agent tris(2-carboxyethyl) phosphine, and that this effect is attenuated by Kvβ3, which was initially identified as a K^+^ channel auxiliary subunit [20]. However, the physiological significance of Panx1 channel redox regulation is unknown. Further studies are needed to understand the control of Panx1 channels under physiological or pathological conditions.

Stomatin-like proteins (SLPs) are characterized by the presence of an evolutionarily conserved core domain known as the stomatin domain. The majority of identified SLPs have a short hydrophobic domain near the amino terminus, which may be used for anchorage to the intracellular side of the plasma membrane through a hairpin structure [21]. There are at least five SLPs in mammals, including stomatin, SLP-1, SLP-2, SLP-3 and podocin [21]. Several of them as well as MEC-2, which is a *C. elegans* SLP, regulate the activities of membrane channels or transporters [22]–[26]. In addition, the *C. elegans* SLP UNC-1 is required for the function of gap junctions formed by the innexin UNC-9, probably through an effect of UNC-1 on gap junction gating [27]. Thus, SLPs appear to play important roles with respect to the functions of membrane channels, transporters, and gap junctions.

The regulation of UNC-9 gap junctions by UNC-1 in invertebrates raised the possibility that gap junctions or hemichannels formed by pannexins are also modulated by SLPs in mammalian system. The present study focused on potential regulation of Panx1 hemichannels by stomatin because both proteins are almost ubiquitously expressed in mammals [1], [3], [28], and Panx1 functions mainly, if not exclusively, as hemichannels in native tissues [29]. We will refer to these channels as Panx1 channels as suggested recently by other investigators [29]. We found that stomatin inhibited Panx1 channel activity when it was co-expressed with Panx1 in HEK-293 cells. Furthermore, analyses of primary cultures of astrocytes, which were chosen because the presence and function of Panx1 channels in these cells are well established [11], [30]–[32], confirmed the importance of endogenous stomatin in regulating Panx1 channels. These observations suggest that stomatin may play an important role in keeping Panx1 channels closed under physiological conditions.

## Materials and Methods

### Molecular Cloning

Panx1 and stomatin were cloned from a mouse hippocampal cDNA library by PCR. DNA sequencing indicated that the cloned Panx1 and stomatin matched NM019482 and AF093620, respectively, at the NCBI databank. Subsequently, the full-length Panx1 and stomatin cDNAs were cloned into specific expression vectors. The plasmids wp870 and wp867 were generated by cloning Panx1 and stomatin into pIRES2-EGFP and pIRES2-mCherry vectors (Clontech), respectively. The plasmids wp956 and wp937 were generated by adding Myc and HA epitopes to the carboxyl termini of Panx1 and stomatin in wp870 and wp867, respectively. The plasmids wp982 and wp981 were generated by cloning Myc-tagged Panx1 (Panx1::Myc) and HA-tagged stomatin (stomatin::HA) into a modified pIRES2-EGFP vector, in which the EGFP coding sequence was deleted to avoid potential complication by EGFP fluorescence in immunostaining experiments. The plasmid wp1057 was made by inserting a CMV promoter and stomatin coding sequence into wp870. The plasmid wp1065 was made by inserting a CMV promoter and frame-shifted stomatin coding sequence into wp870. The plasmid wp956 was modified to generate four different new plasmids encoding Panx1 fragments, including wp1020 for Panx1(1–171)::Myc, wp1021 for Panx1(172–426)::Myc, wp1166 for Panx1(235–426)::Myc, and wp1233 for Panx1(172–234).

### Cell Culture and Transfection

HEK-293 (American Type Culture Collection) and HEK-293T cells (Invitrogen) were maintained with Minimum Essential Medium (MEM) supplemented with heat-inactivated 10% fetal bovine serum (Gibco), penicillin (5 µg/ml), and streptomycin (5 µg/ml). Plasmids were transfected into HEK-293 cells with lipofetamine™ 2000 (Invitrogen, Cat:11668) for electrophysiological and immunostaining experiments or into HEK-293T cells for western blot, coimmunoprecipitation, and surface biotinylation assays.

Primary cultures of cortical astrocytes were obtained from P0-2 neonatal C57BL/6 mice. All procedures were approved by the Institutional Animal Care and Use Committee at the University of Connecticut Health Center (Farmington, CT). Briefly, brains were removed from decapitated mouse pups. After removing all meninges, the cortices were dissected, chopped into fine pieces, and enzymatically digested using a papain neural tissue dissociation kit according to manufacturer’s protocol (Miltenyi Biotec, Auburn, CA). Cells were then cultured in T75 tissue culture flasks (Grenier Bio-One, Germany) in DMEM (Gibco, Carlsbad, CA), supplemented with 10% fetal bovine serum (Atlanta Biologicals, Lawrenceville, GA) and antibiotics (Penicillin/Streptomycin; Invitrogen). After 12–16 hours, all non-adherent cells were removed by complete medium change. Mixed glial cultures were maintained for 2–3 weeks in culture (100% humidity; 95% air, 5% CO2, 37°C), shaken overnight to remove microglia, at which time ∼90–95% of the cells were immunopositive for glial fibrillary acidic protein (GFAP). Astrocytes were transfected with either 50 nM fluorescein-conjugated scrambled siRNA (Santa Cruz, Cat: sc-36869) or 25 nM stomatin siRNA (Santa Cruz, Cat: sc-61621) plus 25 nM fluorescein-conjugated scrambled siRNA, using Lipofectamine™ RNAiMAX (Invitrogen, Cat: 13778) following manufacturer’s protocol. Astrocytes were split on coverslips 30–36 hours after transfection and cultured for additional 12–20 hours. Isolated astrocytes with green fluorescence expression were picked for whole-cell patch clamping.

### Electrophysiology

HEK-293 cells were transfected independently with Panx1 (wp870) plus a mCherry empty vector, Panx1 (wp870) plus stomatin (wp867), stomatin (wp867) plus an EGFP empty vector, and both empty vectors (1 µg of each plasmid DNA in 600 µl transfection reaction). HEK293 cells were split and plated onto glass coverslips 20 hours after transfection whereas mouse astrocytes were split and plated onto glass coverslips 30–36 hours after transfection. Electrophysiological recordings were performed 8–16 hours after the splitting for transfected HEK-293 cells, and 12–20 hours after the splitting for transfected astrocytes. A Nikon E600FN upright microscope with a 40X water-immersion objective (Plan Fluor, NA 0.8) and appropriate fluorescence filter sets was used for visualizing the cells. Transfected cells were identified based on the fluorescence of EGFP, mCherry, or fluorescein. Borosilicate glass pipettes were used as electrodes for recording whole-cell currents in response to a voltage ramp (–60 mV to +90 mV over 10 sec) in the classical whole-cell configuration. The voltage-clamp experiments were performed using a Multiclamp 700A amplifier (Molecular Devices), a digitizer (1440A Digidata, Molecular Devices), and the Clampex software (Version 9, Molecular Devices). Data were sampled at a rate of 10 kHz after filtering at 2 kHz. The extracellular solution contained (in mM) NaCl 147, HEPES 10, glucose 13, CaCl_2_ 2, MgCl_2_ 1, and KCl 2 (pH 7.35). The pipette solution contained (in mM) Cs-gluconate 147, HEPES 10, EGTA 10, and MgCl_2_ 3 (pH 7.25). Current density was calculated as the ratio of whole-cell current amplitude over membrane capacitance. The Panx1 mimetic blocking peptide ^10^Panx1 (WRQAAFVDSY) was from AnaSpec (61911).

**Figure 1:**
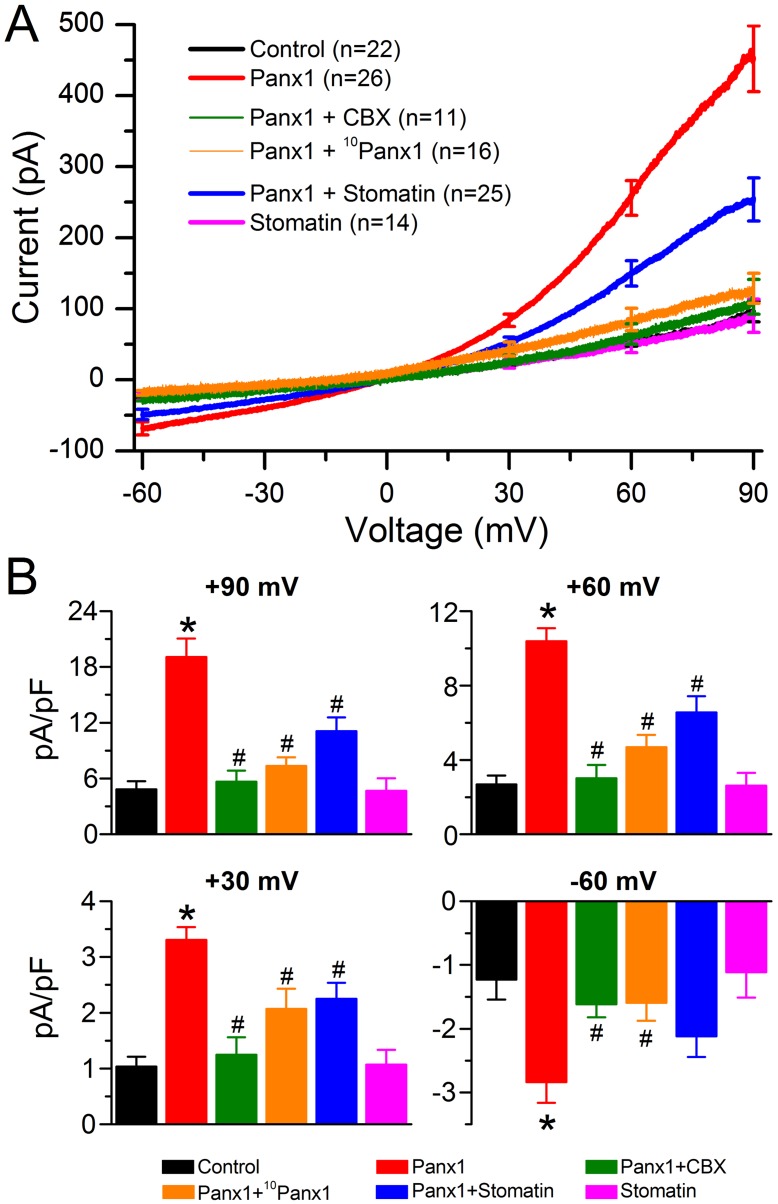
Stomatin inhibited Panx1 channel-mediated outward whole-cell currents in transfected HEK-293 cells. HEK-293 cells were transfected independently with Panx1, stomatin, Panx1 plus stomatin, and empty vectors (Control). Whole-cell currents in response to a voltage ramp (–60 to +90 mV over 10 sec) were recorded from the transfected cells either in the absence or presence of the hemichannels blockers carbenoxolone (CBX 25 µM) or ^10^Panx1 (100 µM). **A.** Averaged current traces in response to a voltage ramp (–60 to +90 mV over 10 sec). Cells expressing Panx1 showed large voltage-dependent outward currents compared with the Control, which were blocked by either CBX or ^10^Panx1 and reduced by stomatin. Note that the Control current trace is almost invisible due to overlap with other traces. **B.** Statistical comparisons of the whole-cell current density at indicated membrane voltages. The asterisk (*) indicates a significant difference compared with the Control whereas the pound sign (#) indicates a significant difference compared with the Panx1 group (*p*<0.01, one-way ANOVA with Bonferroni posthoc tests).

### Dye Uptake

HEK-293 cells were transfected independently with four different plasmids, including Panx1 plus stomatin (wp1057), Panx1 plus frame-shifted stomatin (wp1065), stomatin alone (wp907), and an empty vector. After splitting the cells into low density, dye uptake experiments were performed either with or without whole-cell voltage clamp. In the experiments without whole-cell voltage clamp, either PBS (KH_2_PO_4_ 1 mM, NaCl 155 mM, Na_2_HPO_4_ 3 mM, pH 7.4, Invitrogen) or a high K^+^ solution (KCl 150 mM, HEPES 10 mM, glucose 13 mM, CaCl_2_ 2 mM, MgCl_2_ 1 mM, pH 7.35) containing ethidium bromide (20 µM) was applied to the cells after the cells had been washed with PBS for two times. Fluorescence images were taken with a cooled digital camera (F-view II, Olympus) mounted on an inverted microscope (TE2000-U, Nikon) equipped with a 20X CFI Plan Apo objective (NA 0.75) and appropriate filter sets (FITC and Texas Red). The fluorescence intensity of transfected cells, as identified by EGFP expression, at 5 min (200 ms exposure time) was background subtracted using Image J software (http://rsb.info.nih.gov/ij/) and used for statistical comparisons. In the experiments with whole-cell voltage clamp, a cover glass containing transfected cells was placed in the recording chamber. After perfusing the chamber with 5–10 volumes of extracellular solution, one transfected cell was identified based on EGFP fluorescence and voltage-clamped using the classical whole-cell configuration. A concentrated solution of ethidium bromide was pipetted into the recording chamber to reach a final concentration of 20 µM while the cell was held at –60 mV. A fluorescence image was taken immediately followed by clamping the cell to +80 mV in 5-sec pulses separated by 1.5-sec pulses to –60 mV for 2 min. The –60 mV pulses helped to maintain the whole-cell configuration. A second fluorescence image was taken immediately at the termination of the 2-min voltage clamp protocol. Fluorescence intensity of the cell was background subtracted for comparisons between the two acquired images. The fluorescent images were taken using an electron-multiplying CCD camera (iXonEM+885, Andor Technology, Belfast, Northern Ireland), a FITC filter set (59222, Chroma Technology Corp., Bellows Falls, VT, USA), a light source (Lambda XL, Sutter Instrument, Novato, CA, USA), and the NIS-Elements software (Nikon). Imaging was coordinated with the voltage clamp protocol through a TTL signal between the camera and the digitizer 1440A. The microscope objective and solutions used were identical to those described for the other electrophysiological experiments (see above). Only one cell was used for each cover glass of transfected cells.

**Figure 2:**
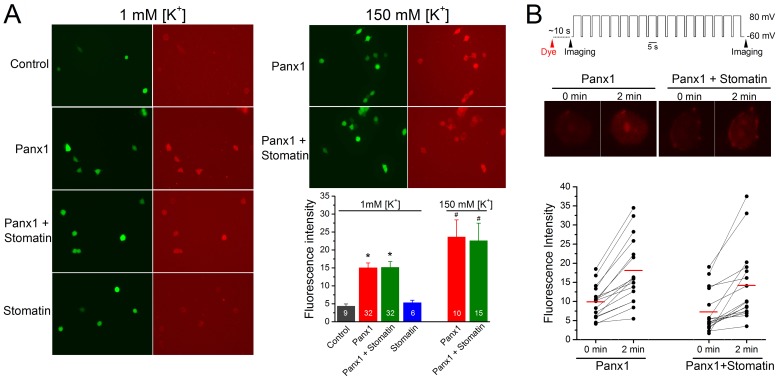
Stomatin did not inhibit Panx1-dependent ethidium uptake in transfected HEK-293 cells. HEK-293 cells were transfected with Panx1, stomatin, Panx1 plus stomatin, or empty vectors (Control). **A.** Transfected cells were incubated with either phosphate-buffered saline (1 mM K^+^) or 150-mM [K^+^] solution containing ethidium bromide (20 µM) for 5 min. Fluorescence intensity of the cells (identified by GFP fluorescence) at 5 min of the incubation was background subtracted and compared among the different groups. The asterisk (*) indicates significant difference compared with the Control while the pound sign (#) indicate significant difference compared with the Panx1 group at 1 mM [K^+^]_o_ (*p*<0.01, one-way ANOVA with Bonferroni posthoc tests). The numbers inside the columns indicate the number of cells analyzed. **B.** Transfected cells were voltage-clamped to +80 mV (5 sec) and –60 mV (1.5 sec) alternatively for a total duration of 2 min immediately following the addition of ethidium (20 µM) to the bath solution. The brief sojourns to –60 mV were necessary for maintaining the whole-cell configuration. Fluorescence intensities of the cells at the beginning and end of the voltage pulses were imaged. The red horizontal lines in the graph indicate the means. Fluorescence intensities are shown in arbitrary units in the plots of both A and B.

### Coimmunoprecipitation

HEK-293T cells were independently transfected with the plasmids encoding HA-tagged stomatin (wp937) and Myc-tagged Panx1 of either full-length (wp956) or variant fragments (wp1020, 1021, and 1166). Cells were harvested 48 hours after transfection and lysed in a buffer containing 1% CHAPS detergent (3-[(3-cholamidopropyl)dimethylammonio]-1-propanesulfonate), 150 mM NaCl, 1 mM CaCl_2_, and 62.5 mM Tris (pH 6.8) supplemented with a protease inhibitor cocktail (Roche, 11836170001). The supernatants of the cell lysates were incubated with a Myc antibody (Santa Cruz, SC-40) at 4°C for 2 hours and immunoprecipitated with protein A/G PLUS agarose (Santa Cruz, SC-2003) at 4°C for 90 min. Immune complexes were separated on 8–16% SDS-PAGE gels and probed with a HA antibody (Santa Cruz, SC-7392).

### Surface Biotinylation

Biotinylation assays were performed using the Cell Surface Protein Isolation kit (Pierce, 89881). Surface proteins were biotinylated 48 hours after the transfection, precipitated with neutrAvidin–agarose beads, and eluted with SDS sample buffer (1% SDS, 50 mM DTT, 10% glycerol, and 62.5 mM Tris, pH 6.8). Total lysate or biotinylated proteins were separated by 4–12% SDS-PAGE, and the blots were detected as described above. The intensities of specific protein bands were determined using the Image J software.

### Immunocytochemistry

Forty-eight hours after transfection, HEK-293 cells coexpressing Panx1::Myc (wp982) and stomatin::HA (wp981) were fixed in 4% paraformaldehyde, permeabilized with 0.1% Triton X-100, blocked with 5% donkey serum, and incubated with mouse Myc and rabbit HA antibodies (Santa Cruz, sc-40; Thermo Scientific, RB-1438-P0). The primary antibodies were detected with Alex Fluor 488-conjugated goat anti-mouse (Molecular Probes, A-11001) and Alex Fluor 594−conjugated goat anti-rabbit secondary antibodies (Molecular Probes, A-11012). Fluorescence images were obtained with a Zeiss LSM 510 Meta confocal microscope.

**Figure 3:**
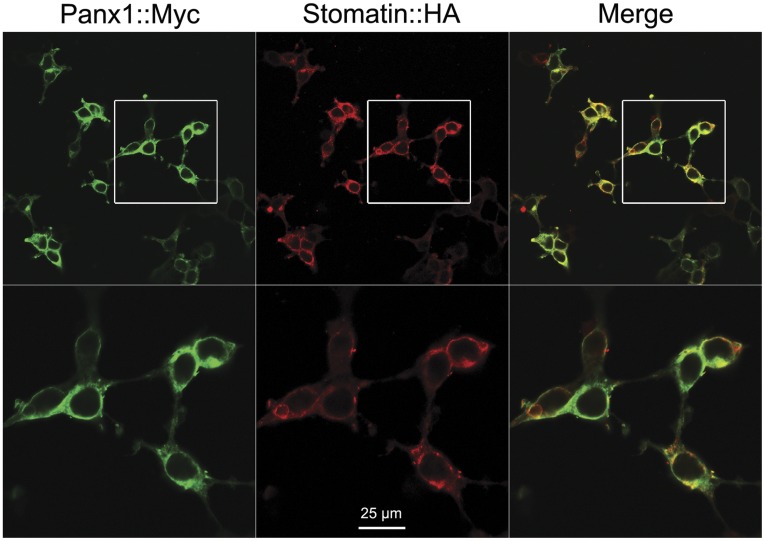
Stomatin and Panx1 were both enriched in the plasma membrane region. Myc-tagged Panx1 (Panx1::Myc) and HA-tagged stomatin (Stomatin::HA) were coexpressed in HEK-293 cells. Panx1 and stomatin were visualized by double immunostaining with antibodies to Myc and HA, respectively. Shown are representative images of Panx1::Myc (green), Stomatin::HA (red), and the merged picture. A selected region in the top panel was magnified and shown in the bottom panel.

### Quantitative PCR (qPCR)

Total RNA was isolated from cultured mouse astrocytes using TRIzol® reagent (Invitrogen) for synthesizing first-strand cDNA. The level of stomatin mRNA was determined by qPCR using SsoFast™ EvaGreen® Supermix (Bio-Rad, 172–5200) and stomatin-specific primers (sense, GTGCACTGACAGCCTCATCAA; antisense, AGCGCATTCCTGAGGGTAGTT). Samples were analyzed in triplicate and normalized by GAPDH expression level, which was detected using specific primers (sense, ACCACCATGGAGAAGGC; antisense, GGCATGGACTGTGGTCATGA). The qPCR was performed with Mastercycler® ep Realplex^2^ (Eppendorf) and analyzed using the 2^−ΔΔCt^ method normalizing to GAPDH expression levels [33].

### Data Analyses

Data are shown as mean ± SE. Statistical analyses and data graphing were performed with Origin 8 (OriginLab, Northampto, MA). Either *t*-tests or ANOVA (with Bonferroni posthoc tests), as specified in figure legends, were used for statistical comparisons. *p*<0.05 is considered to be statistically significant.

**Figure 4:**
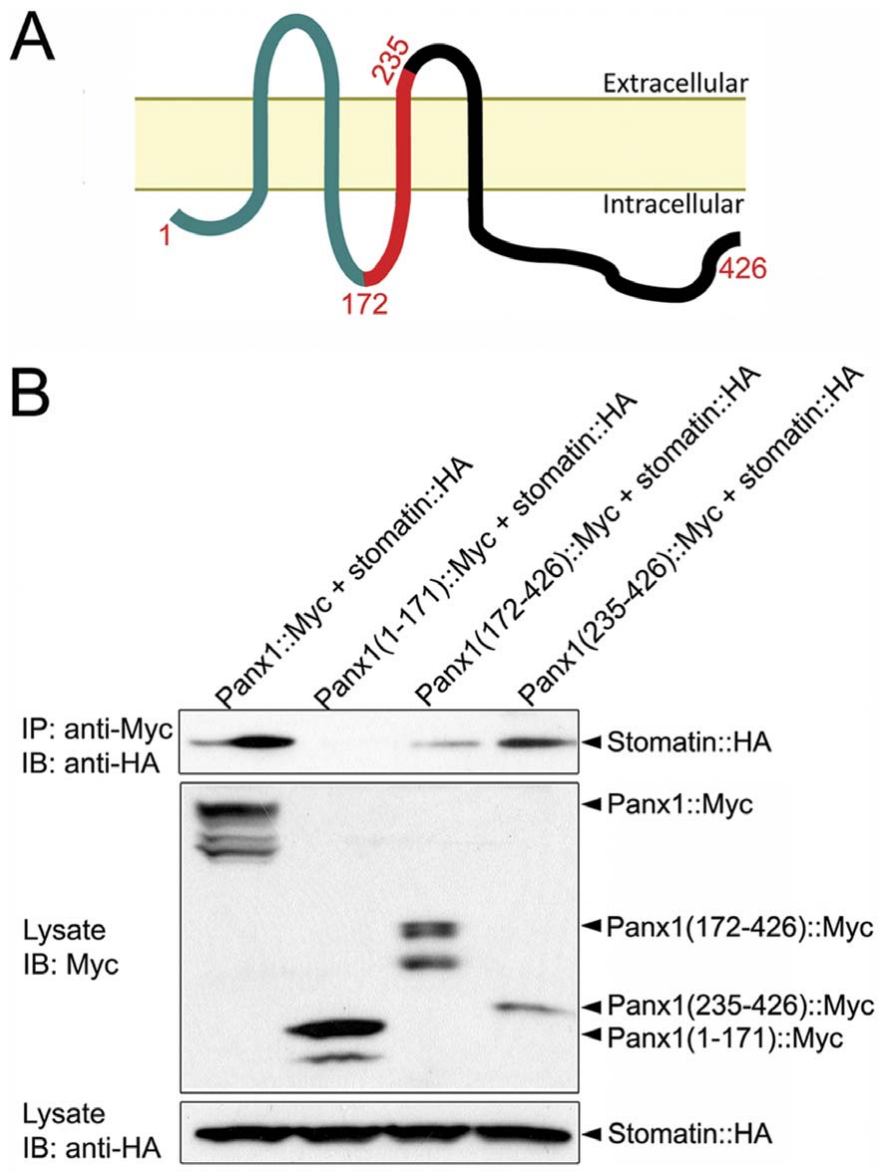
Stomatin coimmunoprecipitated with Panx1. HA-tagged stomatin (Stomatin::HA) was coexpressed with Myc-tagged Panx1 (Panx1::Myc) of either the full-length or a fragment in HEK-293T cells. **A.** Diagram of Panx1 membrane topology. The numbers indicate the positions where truncation or deletion was made. **B.** *Top:* Protein-protein interactions were assessed by immunoprecipitating (IP) with a Myc antibody followed by immunoblotting (IB) with a HA antibody. Stomatin co-imunoprecipitated with full-length Panx1, Panx1(172–426), and Panx1(235–426) but not Panx1(1–171). *Middle and Bottom:* Immunoblots for Panx1 (full-length and fragments) and stomatin in whole-cell lysate. The multiband migrating pattern of Panx1 presumably resulted from either glycosylation or phosphorylation.

## Results

### Stomatin Inhibited Panx1-mediated Whole-cell Currents in HEK-293 Cells

To determine whether stomatin modulates Panx1 channels, we expressed Panx1 and stomatin either separately or in combination in HEK-293 cells, and recorded whole-cell currents in response to a voltage ramp. In the control group (transfected with empty vectors), small inward and outward currents were observed in response to the voltage ramp (Figure 1). Cells expressing Panx1 showed larger currents compared with the control with the difference being more obvious in the positive voltage range (Figure 1A). The difference was statistically significant at all the selected voltages analyzed (–60, +30, +60, and +90 mV) (Figure 1B). Pre-incubation with the non-specific gap junction/hemichannel blocker carbenoxolone (CBX, 25 µM) [13], [34] or the specific peptidic Panx1 channel blocker ^10^Panx1 (100 µM) [9], [16] either eliminated or greatly reduced the effect of Panx1 expression on the outward current (Figure 1), suggesting that the current was conducted by Panx1 channels. These results are similar to those of a previous study [16]. Coexpression with stomatin significantly reduced whole-cell outward current at all the analyzed depolarizing voltages (+30, +60, and +90 mV) but had no obvious effect on the inward current (Figure 1). There were two possible causes for the lack of a significant effect of stomatin on inward current: (1) the effect of stomatin was voltage-dependent, and (2) the small amplitude of Panx1 current and sampling errors obscured an inhibitory effect of stomatin. It is difficult to distinguish between these two possibilities based on the existing data. These observations indicate that stomatin is an inhibitor of Panx1 channels, at least at inside positive membrane voltages.

### Stomatin did not Regulate Panx1 Channel-mediated Dye Uptake

Dye uptake is frequently used as an assay for Panx1 channel function, because small fluorescent molecules, such as ethidium and YO-PRO-1, may pass through Panx1 channels [12], [16], [30]. To obtain further evidence regarding the regulation of Panx1 channels by stomatin, we tested the effect of stomatin on Panx1-mediated uptake of ethidium in transfected HEK-293 cells. Transfected cells were identified based on the fluorescence of EGFP marker. We first performed the assay using standard phosphate buffered saline (PBS) containing 1 mM K^+^ as the extracellular solution. Compared with the control (cells transfected with empty vectors), dye uptake was unchanged in cells expressing stomatin alone but greatly increased in cells expressing Panx1 alone (Figure 2A). Cells coexpressing stomatin and Panx1 showed similar dye uptake as cells expressing Panx1 alone, suggesting that stomatin did not regulate Panx1-mediated dye uptake. With the use of PBS as the extracellular solution, the membrane potential was expected to be hyperpolarized. Because the inhibitory effect of stomatin on Panx1-mediated whole-cell currents was only obvious at positive membrane potentials, we also examined the effect of stomatin on ethidium uptake under experimental conditions when the membrane potential was either near 0 mV (by including 150 mM [K^+^] in the extracellular solution) or at +80 mV (by whole-cell voltage clamp). Stomatin did not show an inhibitory effect on Panx1-mediated dye uptake under either experimental condition (Figure 2A, B). Thus, all of the observations suggest that stomatin does not regulate Panx1-mediated dye uptake.

**Figure 5:**
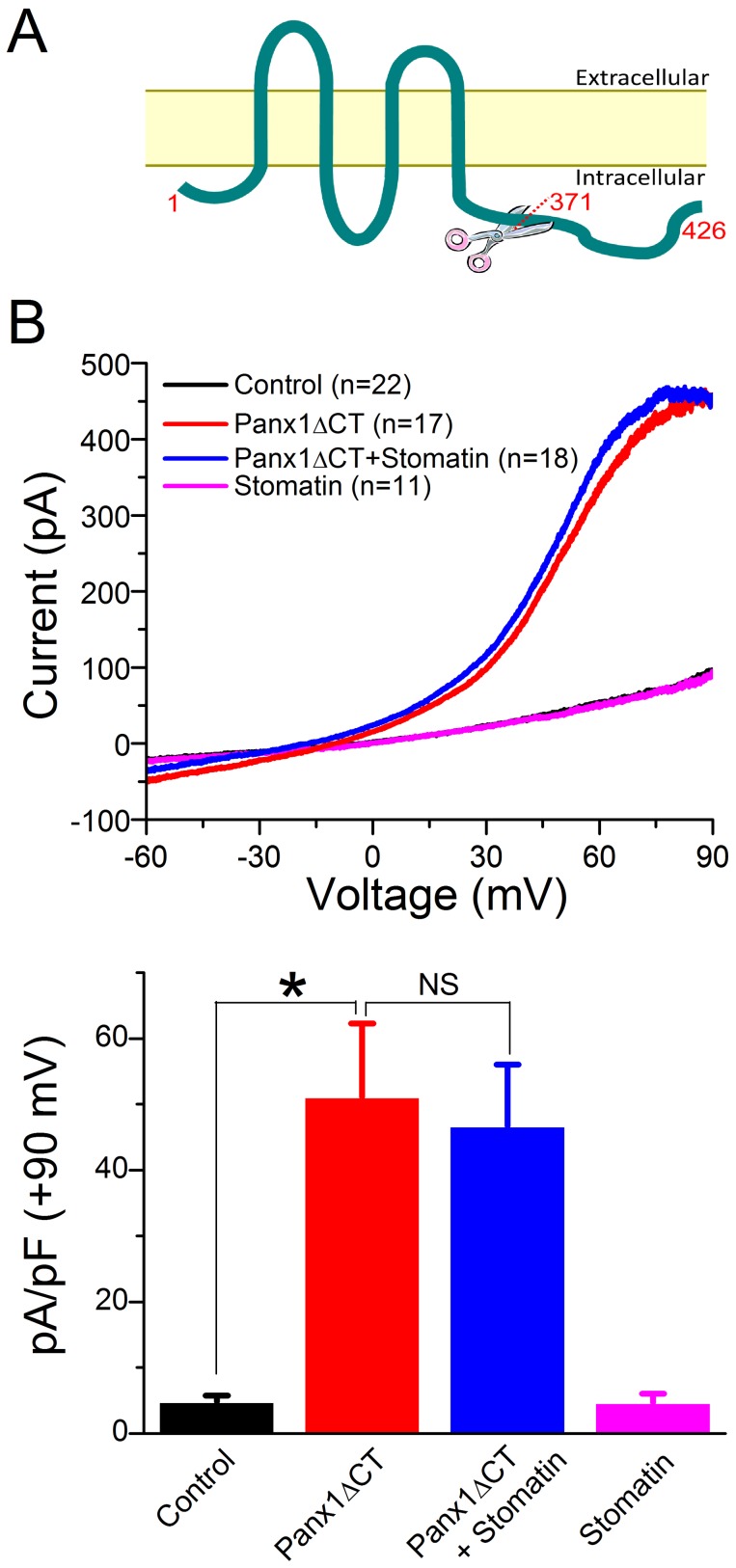
Truncation of the Panx1 carboxyl terminal abolished the inhibitory effect of stomatin. A. Diagram of Panx1 showing the site of carboxyl terminal truncation. **B.** Top: Averaged whole-cell current traces in response to voltage ramps (–60 to +90 mV over 10 sec). HEK-293 cells had been transfected with either empty vectors (Control) or plasmids encoding the truncated Panx1 and/or stomatin. The current trace of Control overlapped with that of Stomatin. Bottom: Comparisons of whole-cell current at +90 mV among the different groups. The asterisk (*) indicates a significant difference compared with the Control (*p*<0.01, ANOVA with Bonferroni posthoc test) whereas “NS” means no significant difference.

**Figure 6:**
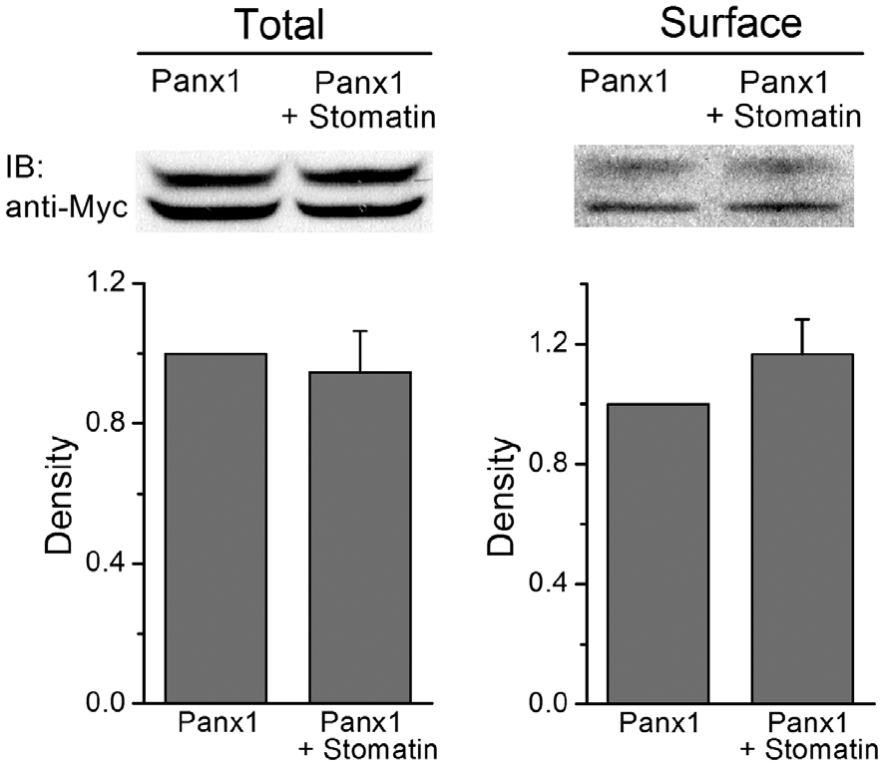
Stomatin did not alter total or cell surface expression of Panx1. The amount of total and surface Panx1 protein levels were determined with transfected HEK-293 cells expressing either Myc-tagged Panx1 (Panx1::Myc) alone or Panx1::Myc plus HA-tagged stomatin (Stomatin::HA). The bar graphs show the densities of the Panx1::Myc total and surface protein bands (normalized by actin) based on 5 independent experiments.

### Stomatin and Panx1 were Enriched in the Plasma Membrane

Panx1 functions in the plasma membrane to conduct currents. The inhibition of Panx1-mediated outward currents by stomatin suggested that these two proteins likely colocalize in the plasma membrane. To examine this possibility, we fused Myc and HA to the carboxyl termini of Panx1 and stomatin, respectively, and analyzed subcellular localization of these two fusion proteins in transfected HEK-293 cells by double-immunostaining with antibodies specific to Myc and HA. Both fusion proteins were enriched in the plasma membrane region with intracellular expression also detected (Figure 3), which are similar to previous reports about Panx1 [16], [35] and stomatin [36]–[38]. The observed subcellular localization patterns of stomatin and Panx1 are consistent with the regulatory effect of stomatin on Panx1 channels (Figure 1) and the surface biotinylation data of Panx1 (Figure 6).

### Stomatin Physically Interacted with Panx1

The modulatory effect of stomatin on Panx1 currents and their colocalization in the plasma membrane suggest that these two proteins might interact physically. To examine this possibility, we performed coimmuneprecipitation experiments with Myc-tagged Panx1 (Panx1::Myc) and HA-tagged stomatin (stomatin::HA) coexpressed in HEK-293 cells. Panx1 is predicted to have four transmembrane domains, two extracellular loops, and one intracellular loop with both the amino and carboxyl termini located on the intracellular side (Figure 4A) [15], [39]. Full-length Panx1 coimmunoprecipitated with full-length stomatin (Figure 4B), suggesting that these two proteins existed in the same molecular complex. To determine which part of Panx1 is important to its physical interaction with stomatin, we constructed three different Myc-tagged Panx1 fragments, including Panx1(1–171), Panx1(172–426), and Panx1(235–426). Panx1(1–171) and Panx1(172–426) corresponded to the amino and carboxyl terminal portions of Panx1 divided at the intracellular loop while Panx1(235–426) consisted of a portion of the second extracellular loop, the fourth membrane-spanning domain, and the cytosolic carboxyl terminal (Figure 4A). We found that either Panx1(172–426) or Panx1(235–426) coimmunoprecipitated with stomatin whereas Panx1(1–171) did not (Figure 4B), suggesting that the carboxyl terminal portion of Panx1 plays a critical role in interacting with stomatin. Intriguingly, the shorter Panx1(235–426) appeared to have a stronger interaction with stomatin than the longer Panx1(172–426). To determine how the additional sequence in Panx1(172–426) might have affected the coimmunoprecipitation experiment, we attempted to express Myc-tagged Panx1(171–234) as a separate protein in HEK-293 cells. However, western blot did not detect any expression of this Panx1 fragment in transfected cells, which made further analyses difficult.

**Figure 7:**
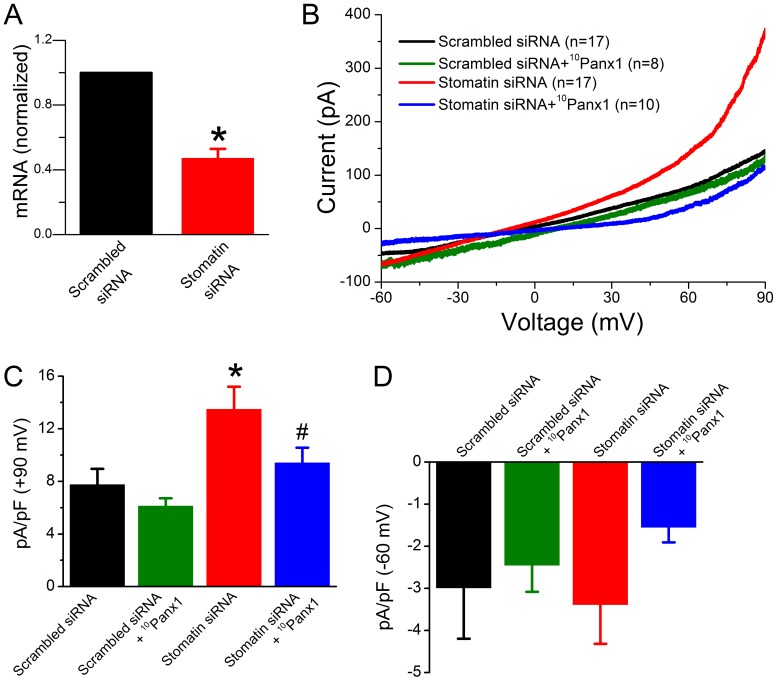
Inhibition of stomatin expression enhanced Panx1-dependent outward whole-cell currents in astrocytes. Cells were transfected with either stomatin siRNA or scrambled siRNA. **A.** Effects of scrambled and stomatin siRNA on stomatin mRNA level as determined by real-time PCR (from 3 independent experiments). The asterisk (*) indicates a significant difference compared with scrambled siRNA (*p*<0.01, paired *t*-test). **B.** Averaged current traces in response to a voltage ramp (–60 to +90 mV over 10 sec) showing the effects of siRNA and the specific Panx1 channel inhibitor ^10^Panx1. **C & D.** Comparisons of the peak outward (at +90 mV) and inward (at –60 mV) currents among the different groups. The asterisk (*) indicates a significant difference (*p*<0.01) compared with scrambled siRNA whereas the pound sign (#) indicates a significant difference (*p*<0.05) compared with stomatin siRNA (one-way ANOVA with Bonferroni posthoc test).

### Panx1 Carboxyl Terminal was Required for the Regulation by Stomatin

It has been shown that a truncated Panx1, in which the cytosolic carboxyl terminal starting from the amino acid residue 372 is deleted, can form membrane channels that are more active than those formed by full-length Paxn1 [12]. To better understand the role of Panx1 carboxyl terminal in the regulation by stomatin, we expressed Panx1ΔCT (Panx1 amino acids 1–371) in HEK-293 cells, and tested the effect of stomatin on channels formed by the truncated protein. In response to the voltage ramp, the density of the peak outward currents (at +90 mV) mediated by Panx1ΔCT (Figure 5) was 3-fold larger than that mediated by full-length Panx1 (Figure 1). Because cells expressing Panx1ΔCT were much smaller than those expressing full-length Panx1, as indicated by visual inspection and measurement of cell capacitance, the amplitude of outward currents at +90 mV was comparable between the full-length Panx1 and Panx1ΔCT groups (Figures 1A and 5B). Unlike the full-length Panx1, the Panx1ΔCT-mediated outward currents were not inhibited by stomatin (Figure 5), suggesting that the presence of Panx1 carboxyl terminal was essential to the regulation by stomatin.

### Stomatin did not Affect Panx1 Total or Surface Protein Level

The inhibitory effect of stomatin on Panx1 channel currents could be due to either an inhibition of Panx1 channel function or a down-regulation of Panx1 protein level. To examine the second possibility, we analyzed the total and surface protein levels of Panx1 with homogenates of HEK-293 cells transfected with either Panx1 alone or Panx1 plus stomatin. Both the total and surface Panx1 protein levels were comparable between the cells with and without stomatin (Figure 6). These observations suggest that the inhibitory effect of stomatin on Panx1 channels unlikely resulted from a change in Panx1 protein translation, stability or membrane trafficking; instead stomatin might modulate the function of Panx1 channels.

### Inhibition of Stomatin in Mouse Astrocytes Resulted in Increased Panx1 Currents

The analyses with HEK-293 cells suggested that stomatin is potentially a physiological regulator of Panx1 channels in native tissues. We explored this possibility with primary culture of mouse astrocytes because these cells have Panx1 hemichannels in the plasma membrane [11], [30]–[32] and potentially express stomatin. The role of endogenous stomatin was assessed by analyzing the effect of stomatin siRNA on whole-cell currents in response to a voltage ramp. Quantitative PCR (qPCR) and electrophysiological analyses were performed 48–72 hours after the transfection. Astrocytes subjected to stomatin siRNA treatment showed 53% decrease in stomatin mRNA level (Figure 7A), which was likely an underestimate for transfected cells because the transfection efficiency was approximately 70%. The stomatin siRNA significantly increased outward currents in response to the voltage ramp compared with the scrambled siRNA (Figure 7B, C). Application of ^10^Panx1 (150 µM) abolished the augmenting effect of stomatin siRNA on outward whole-cell currents but had no effect in astrocytes transfected with scrambled siRNA (Figure 7B and C). Intriguingly, stomatin siRNA treatment changed the slope of the averaged current trace whether or not ^10^Panx1 was present. This effect of stomatin siRNA might reflect reduction in inhibition of another channel(s). These observations suggest that endogenous stomatin likely plays an important role in regulation of endogenous Panx1 channels in astrocytes.

## Discussion

In the present study, we identified stomatin as a novel inhibitor of Panx1 channels. This conclusion was supported by data from HEK-293 cells transfected with stomatin and Panx1 as well as mouse astrocytes expressing endogenous stomatin and Panx1. Given that both proteins are almost ubiquitously expressed in mammals, stomatin potentially plays an important role in regulating the physiological function of Panx1 channels in many other cells as well.

How might stomatin regulate the function of Panx1 channels? The lack of effects of stomatin on Panx1 total and surface protein levels suggests that stomatin did not inhibit Panx1 channels through reducing Panx1 transcription, translation, membrane trafficking, or protein stability. As stomatin is an integral membrane protein [21] whose distribution overlapped with that of Panx1 in transfected HEK-293 cells (Figure 3), it may well regulate Panx1 channels through a direct interaction. Previous studies suggest that SLPs regulate gap junctions and mechanosensitive channels through effects on channel gating [22], [24], [27], [40]. This could be also the case with respect to the regulation of Panx1 channels by stomatin. Because stomatin coimmunoprecipitated with Panx1 carboxyl terminal and its inhibitory effect on Panx1 channels was abolished by deleting the carboxyl terminal of Panx1, stomatin probably regulates channel gating through interacting with the Panx1 carboxyl terminal. This notion is compatible with the previous observation that appending GFP to the carboxyl terminus of Panx1 reduces Panx1 channel currents [41] and appending GFP to the carboxyl terminus of the innexin UNC-9 makes the function of UNC-9 independent of the SLP UNC-1 [27].

Genetic and electrophysiological work with *C. elegans* suggests that interactions between SLPs and gap junctions or mechanosensitive channels are specific. While there are 10 SLPs, 25 innexins, and a variety of mechanosensitive ion channels in *C. elegans* (www.wormbase.org), only two SLPs (UNC-1 and UNC-24) have been implicated in the functions of two innexins (UNC-7 and UNC-9) [27], [42]–[48], and only one SLP (MEC-2) modulates the function of mechanosensitive channels formed by the degenerin/epithelial Na^+^ channel proteins MEC-4 and MEC-10 [22]. Given that there are 5 SLPs [21] and 3 pannexins in mammals [1], potential regulations of pannexin hemichannels or gap junctions by SLPs might also involve protein-specific interactions.

Panx1 channel activities are reportedly associated with increases in both membrane currents and permeability to fluorescent dyes [12], [13], [16], [30]. In the present study, however, stomatin showed differential effects on Panx1-dependent whole-cell currents and dye uptake. While stomatin inhibited Panx1-dependent outward whole-cell currents, it did not affect ethidium uptake by HEK-293 cells transfected with Panx1. This disparity did not appear to be due to differences in the membrane voltage because the effect of stomatin on dye uptake was analyzed under three different conditions. Studies of gap junctions formed by various connexins have shown that pore size or single channel conductance often does not correlate with selectivity or dye permeability, and that dye permeability is often at least of 2 orders of magnitude larger than predicted on the basis of pore diffusion [49]–[51]. It has been suggested that an affinity between the dye and pore underlies the large dye permeability [51]. Conceivably, ionic current and dye flux through Panx1 channels might depend on distinct molecular and/or gating mechanisms, and stomatin was effective in only regulating current flow.

The finding that stomatin regulates Panx1 channels potentially has wide implications. For example, stomatin might play a protective role against hypoxia- or ischemia-induced cell damage because hypoxia-induced elevation of stomatin expression [52] could counteract hypoxia-induced increase of Panx1 channel activity [13]. Stomatin might also regulate membrane permeability of erythrocytes by modulating Panx1 channels since Panx1 channels are present in erythrocyte plasma membrane [4], [17] and stomatin disappears from the membrane in patients with the hemolytic disease stomatocytosis [38]. Stomatin might modulate neuronal epileptiform seizure activity and astrocytic protection for neurons through Panx1 channels [9], [10]. Given that there are multiple SLPs and pannexins in mammals, hemichannels or gap junctions formed by other pannexins might also be regulated by SLPs. Furthermore, although connexins do not belong to the same family of proteins as pannexins, hemichannels and gap junctions formed by them are often modulated by similar physiological and pharmacological factors. Therefore, it will be interesting to investigate whether SLPs also regulate hemichannels or gap junctions formed by connexins and other pannexins or innexins.
